# Susceptibility of *Myzus persicae*, *Brevicoryne brassicae* and *Nasonovia ribisnigri* to Fungal Biopesticides in Laboratory and Field Experiments

**DOI:** 10.3390/insects11010055

**Published:** 2020-01-17

**Authors:** Gill Prince, Dave Chandler

**Affiliations:** Warwick Crop Centre, School of Life Sciences, University of Warwick, Wellesbourne, Warwick CV35 9EF, UK; g.prince@warwick.ac.uk

**Keywords:** integrated pest management, entomopathogenic fungi, aphids, vegetables, horticulture

## Abstract

The aim of this study was to evaluate the potential of entomopathogenic fungi (EPF) for the control of aphid pests of field vegetable crops. Four biopesticides based on the EPF *Beauveria bassiana* (Botanigard ES and Naturalis L), *Cordyceps fumosorosea* s.l. (Preferal WG), and *Akanthomyces dipterigenus* (Vertalec) were evaluated in a laboratory bioassay against peach-potato aphid *Myzus persicae*, cabbage aphid *Brevicoryne brassicae*, and currant-lettuce aphid *Nasonovia* *ribisnigri*. There was significant variation in the spore dose provided by the products, with Botanigard ES producing the highest dose (639 viable spores per mm^2^). Botanigard ES also caused more mortality than the other products. Combining Vertalec with the vegetable oil-based adjuvant Addit had an additive effect on the mortality of *B.* *brassicae*. All fungal products reduced the number of progeny produced by *M. persicae* but there was no effect with *B. brassicae* or *N. ribisnigri*. When aphid nymphs were treated with Botanigard ES and Preferal WG, both products reduced population development, with up to 86% reduction occurring for Botanigard ES against *M. persicae*. In a field experiment, Botanigard ES sprayed twice, at seven-day intervals, against *B. brassicae* on cabbage plants, reduced aphid numbers by 73%. In a second field experiment with *B. brassicae*, *M. persicae*, and *N. ribisnigri*, Botanigard ES reduced populations of *B. brassicae* and *N. ribisnigri* but there was no significant effect on *M. persicae*.

## 1. Introduction

Aphids (Hemiptera: Aphididae) are damaging pests of a wide range of horticultural crops [1,2]. A combination of parthenogenetic reproduction, high fecundity and a short development time mean that aphid populations can build up rapidly on summer host plants [3]. Aphid infestations cause stunted and distorted plant growth, while sooty moulds grow on aphid honeydew deposited on the plant surface—both of which reduce the marketability of fresh produce [4,5]. Many, but not all, aphid species are also vectors of plant pathogenic viruses, which can result in large yield reductions [6,7,8].

This paper concerns three different aphid pests of field vegetable crops. The peach potato aphid, *Myzus persicae* Sulzer, 1776, is a generalist species that causes economic losses on many field-grown horticultural crops including vegetable brassicas and lettuce. It is also a vector of over 100 plant viruses including in many important crops [9]. The cabbage aphid, *Brevicoryne brassicae* Linnaeus, 1758, is a specialist feeder on brassicas and its close relatives and is economically one of the most important insect pests for vegetable brassica growers [2,10,11]. The currant-lettuce aphid, *Nasonovia ribisnigri* Mosley, 1841, utilizes members of the Asteraceae as secondary (i.e., summer) hosts and is a serious problem for lettuce production in northern Europe [2,12,13,14].

For many years, the standard method for controlling aphids on field vegetables has been through foliar sprays of synthetic chemical pesticides [14,15,16]. However, growers are under pressure to reduce their use of toxicant pesticides because of concerns about residues in food, as well as increasing recognition of the need to reduce the harmful effects of pesticide applications on beneficial insects and other non-target organisms [17,18,19]. In the EU, an increasingly stringent pesticide regulatory system [20] is leading to withdrawals of older products and has placed greater restrictions on the number and timing of spray applications for approved pesticides. There are also long-standing concerns about the evolution of heritable insecticide resistance in aphid populations [21]. *Myzus persicae* is particularly challenging to control because clones with pesticide resistance are now widespread including those with both modified acetylcholinesterase (MACE) resistance to carbamates and knockdown (kdr and super-kdr) resistance to pyrethroid insecticides [22]. Strains of *N. ribisnigri* have also been reported to show resistance to pirimicarb and pyrethroid insecticides [14,23]. Pesticide resistance in *B. brassicae* has not yet been a problem in Europe, although resistance has been reported elsewhere [24] and there is a risk that over-reliance on a small number of pesticide types will increase the selection pressure on resistance evolving. The introduction of neonicotinoids in the late 1990s got around some of the problems with resistance to pyrethroids and other active substances, but recent government restrictions on neonicotinoids on field crops has left farmers and growers with few effective controls. Moreover, the lack of alternatives to synthetic chemical pesticides for aphid control on field vegetables is preventing management moving onto a more sustainable footing as part of Integrated Pest Management (IPM) [25].

One option for an alternative method of control is the use of microbial biopesticides based on entomopathogens. Entomopathogenic fungi (EPF), which infect their hosts percutaneously, are potentially the best candidates against aphids and other sap-feeding insects, which are difficult to treat with entomopathogens that infect by ingestion, such as viruses and bacteria [26]. They are also considered to present minimal risk to non-target, beneficial organisms including natural enemies and as such make good components of IPM [27]. An effective EPF biopesticide might be able to stop the population development of aphids on field vegetable crops and reduce the level of plant damage in an environmentally benign way. Worldwide, over 170 EPF biopesticide products have been developed [28]. In Europe, a number of products are available against foliar pests in greenhouses, where environmental conditions are considered conducive to EPF activity [29,30,31], and this raises the question of whether they have potential also against aphids on field vegetables. Botanigard^®^, based on *Beauveria bassiana* (Bals.-Criv.) Vuill. 1912 strain GHA (Hypocreales, Cordycipitaceae) is marketed against whiteflies, thrips, aphids and mealybugs [31]. Naturalis-L^®^, based on *B. bassiana* strain JW-1/ATCC 74040, is used against whitefly and thrips [31]. Preferal WG^®^, based on *Cordyceps fumosorosea* s.l. (Wize) Kepler, B. Shrestha and Spatafora, comb. nov. strain PFR-97/Apopka 97 (formerly *Isaria fumosorosea* s.l./*Paecilomyces fumosoroseus*) (Hypocreales, Cordycipitaceae) [32] is sold for whitefly control [31]. A fourth product, Vertalec^®^, based on *Akanthomyces dipterigenus* (Petch) Spatafora, Kepler, Zare and B. Shrestha, comb. nov. strain 1.72 (formerly *Lecanicillium longisporum*/*Verticillium longisporum*) [32] was developed as a biopesticide specifically for greenhouse aphid control [29] and has also been shown to control plant pathogens such as powdery mildews on cucumber in controlled environments [33]. Previous studies have shown it to be effective against *M. persicae*, *N. ribisnigri* and the potato aphid, *Macrosiphum euphorbiae* Thomas, C., 1878, on lettuce crops grown under glasshouse conditions [34]. Over 55 biopesticide products have been developed based on *B. bassiana*, as strains of this species are able to effectively control a wide range of pest insects [28]. *Cordyceps fumosorosea* s.l. also has a relatively large host range as a species, making it attractive for development as a biopesticide product [35], particularly against pests of greenhouse crops [36,37,38].

In this paper, we report on a study of the potential of Botanigard ES, Naturalis-L, Preferal WG and Vertalec to control populations of *M. persicae*, *B. brassicae* and *N. ribisnigri* on brassica and lettuce. The use of EPF against aphids on field vegetable crops in northern temperate areas presents a number of challenges. Good control in the field is reliant on the ability to deliver a lethal dose of spores to the target and requires favourable environmental conditions including suitable temperatures [39]. Many strains of hypocrealean EPF investigated as biopesticides have an optimum temperature of 25 °C or more [40,41,42,43] and in countries such as the UK, where mean summer (day/night) temperatures are consistently lower than this [44], there is a question of whether EPF biopesticide products are able to perform adequately under field temperature conditions. Finally, moulting of nymphs every few days during development may allow aphids to ‘escape’ infection from fungal spores that have adhered to the outer cuticle but have not yet grown and penetrated through to the haemocoel [45]. Therefore, it is important to determine the extent to which nymphs can be controlled by EPF applications in comparison to the effect on adults, including the ability of fungal infection to reduce adult reproduction [46,47]. Our aims were to quantify the effects of commercial EPF biopesticides on adults and nymphs of *M. persicae*, *B. brassicae* and *N. ribisnigri* under laboratory conditions, investigate the effects of EPF on the reproduction of adult aphids, and then quantify the effect of selected EPF on populations of the aphids on brassica and lettuce crops grown in the field. 

## 2. Materials and Methods

### 2.1. Biological Material

The clones of *B. brassicae*, *M. persicae* and *N. ribisnigri* used in the study are shown in Table 1.

Brussels sprouts (*Brassica oleracea*) cv. Montgomery (for *M. persicae*, *B. brassicae*) or lettuce (*Lactuca sativa*) cv. Saladin (for *N. ribisnigri*) seeds were sown in vermiculite. Then after six days, the seedlings were transplanted to Levington F2 compost in 75 mm polyethylene pots and grown on for three weeks (20 ± 2 °C, 60% RH, L:D 16:8 h). Stock cultures of aphids were maintained on 4-week-old plants within mesh cages in a controlled environment room (20 ± 2 °C, 60% RH, L:D 16:8 h). Fixed-age aphid cultures were produced by confining groups of 15 mature apterous virginoparae (female adults without wings which give birth to live young by parthenogenesis) on 4-week-old plants for 24 h—after which, the virginoparae were removed and the progeny maintained for a further seven (*M. persicae*), eight (*N. ribisnigri*) or ten (*B. brassicae*) days. This procedure was repeated up to three times until the number of apterous virginoparae required for experiments was obtained. For laboratory bioassays with EPF, Brussels sprouts cv. Montgomery or lettuce cv. Saladin seeds were sown in vermiculite. Then after six days, the seedlings were transplanted to Levington F2 compost in 75 mm polyethylene pots and grown on for three weeks (20 ± 2 °C, 60% RH, L:D 16:8 h) at which point they were used in the bioassay. Fungal biopesticide products used in the study (Table 2) were obtained from their manufacturers and stored at 4 °C before use. The EPF biopesticide products were prepared in sterile distilled water according to the manufacturers’ instructions. The EPF product Vertalec was also evaluated when applied with each of two vegetable oil-based adjuvants, Codacide (Microcide Ltd., Bury St. Edmunds, UK) and Addit (Koppert BV, Berkel en Rodenrijs, Netherlands). These adjuvants are designed to improve biopesticide adhesion and spread and were applied at the manufacturers’ recommended rate (Table 2).

### 2.2. Laboratory Bioassay of Aphid Susceptibility to EPF Biopesticides

Laboratory bioassays were done to measure the effect of fungal biopesticides on adult aphids of *M. persicae* (clone 4859B), *B. brassicae* (clone K3) and *N. ribisnigri* (clone 4850A) as follows. Groups of 60 apterous virginoparae, taken from a fixed-age culture as described previously, were placed on two sheets of damp filter paper (Whatman #1) within the lid of a 90 mm diameter Petri dish using a fine camel-hair paintbrush. Aphids were then sprayed with 2 mL suspension of each fungal biopesticide using a Potter tower [48] at 50 kPa and left to dry and recover for 1 h. All products were used at the manufacturers’ recommended rate. Groups of 15 individual aphids were then transferred to the base of 3-week-old Brussels sprouts or lettuce plants enclosed within a transparent, low-density polyethylene bakery bread bag (250 × 400 mm with perforations) (Cheverton and Laidler, UK) and secured with two elastic bands. The plants were maintained within a controlled environment room (20 ± 2 °C, 60% RH, L:D 16:8 h) for nine days. The numbers of alive and dead aphids (no movement when the abdomen was lifted repeatedly with the tip of a camel-hair paintbrush) were assessed every 72 h (i.e., day 3, 6 and 9), at which points nymphs were also counted and removed. Any dead adult aphids were removed and incubated on damp filter paper within sealed Petri dishes in an incubator (20 ± 1 °C, darkness) for seven days and inspected for the presence of mycelium on the cadavers. Bioassays using the EPF products, as well as treatments of Vertalec combined with Addit, were repeated on five separate occasions for each aphid species. An additional treatment of Vertalec combined with Codacide was included on the last two occasions. To estimate the dose of EPF (number of spores per mm^2^) sprayed with the Potter tower, a glass coverslip (18 × 18 mm) was placed next to the aphids during spraying: this was then washed in 1 mL 0.05% Triton X-100 to remove spores, and aliquots were serially diluted and plated onto Sabouraud dextrose agar, and then cultured for five days (20 ± 1 °C, darkness)—after which, numbers of colony forming units (cfu) were counted. Percentage mortality data on each plant on days six and nine were corrected for natural mortality in untreated controls [49]. Mortalities of the EPF biopesticide treatments without adjuvants were compared using an ANOVA following a logit transformation, while mortalities associated with Vertalec, Addit and Codacide were compared using a Kruskal Wallis test and Mann Whitney U tests [50]. The outcome of combining Vertalec with Addit or Codacide (synergism, antagonism, or additive effect) was investigated using the fractional product method for combination treatments, where the effect of the combination is given as (1−X), where X = (1 − A)(1 − B) and where A = the proportional effect of agent A on its own, and B is the proportional effect of agent B [51]. The cumulative number of nymphs produced by the adults at days six and nine was analysed using a generalised linear model with a log link function and a Poisson distribution [52]. A second bioassay was also done, using the methods described above, to evaluate the susceptibility of first and second instar nymphs (1–3 days old, reared as described above) to Botanigard ES and Preferal WG. The bioassay was done on one occasion with nine replicate plants per treatment. The effect of the biopesticides was quantified by counting the total number of live aphids on plants on days seven and ten (which therefore included the original cohort of nymphs plus any offspring they had produced at maturity). The data were analysed using a generalized linear model with a log link function and a Poisson distribution [52].

### 2.3. Effect of Beauveria bassiana (Botanigard ES) on Aphid Populations in a Field Experiment

The effect of Botanigard ES on aphid populations was evaluated in a field experiment and compared against an untreated control. The experiment was done on the experimental farm at Warwick Crop Centre, Wellesbourne, Warwickshire UK, and two trials were conducted consecutively, in the same year. On the first occasion, Botanigard ES was evaluated against populations of *B. brassicae* (clone K3) on cabbage plants, while on the second occasion Botanigard ES was evaluated against *B. brassicae* (clone K3, cabbage), *M. persicae* (clone 2050A, cabbage), and *N. ribisnigri* (clone 4850A, lettuce). Each treatment was replicated in four plots; on occasion 2 this was arranged as a split-plot design, with treatment (biopesticide, control) applied to main plots and aphid species applied to sub-plots. For each treatment, plants were grown in beds (5 m long × 1.8 m wide) containing 20 plants (2 rows × 10 plants, plant spacing 50 cm) of cabbage (*B. oleracea*) cv. Montgomery (for *M. persicae* and *B. brassicae*) and lettuce cv. Saladin (for *N. ribisnigri*). For plant raising, cabbage and lettuce seeds were sown in Levington F2 compost and plants grown in a glasshouse compartment at 19 ± 2 °C for 5 weeks—after which, they were transplanted to the field (occasion 1 = 12 July; occasion 2 = 16 August). Each bed was covered in horticultural fleece supported by plastic hoops and with the edges of the fleece buried into the ground. Mature apterous virginoparae of each aphid species (reared as described previously) were then used to inoculate the plants in each bed at a rate of 10 aphids per plant (day zero). For treatment with the biopesticide, the horticultural fleece was temporarily removed and Botanigard ES was applied at the manufacturers recommended rate using a knapsack sprayer equipped with a flat fan nozzle to the foliage of the plants on two occasions; the first at seven days after aphid inoculation and then at 14 days. The fleece was replaced immediately after spraying. The total numbers of aphids on each plant were counted immediately before spraying (i.e., day 7 and 14) and seven days after the second spray (day 21). Mean total number of aphids per plant were compared using an ANOVA on square root-transformed data [52]. Environmental conditions were monitored throughout the experiment from an on-site weather station.

## 3. Results

### 3.1. Laboratory Bioassays

There was significant variation in the mean dose of spores applied (viable spores per mm^2^) for the different EPF biopesticides in the laboratory bioassay, as measured by cfu counts (*F* = 35.88, *d.f. =* 5, *p* < 0.001) (Table 3). In post hoc tests, a larger dose was applied with Botanigard ES compared to the other products (Tukey HSD test; Botanigard ES vs. Preferal WG, *p* = 0.006; Botanigard ES vs. other treatments, *p* < 0.001). Botanigard ES produced a mean dose of 639 viable spores per mm^2^, which was 2.5 times greater than the next highest dose, produced by Preferal WG at 260 spores per mm^2^. Naturalis-L and Vertalec produced markedly lower doses, at less than 70 spores per mm^2^ each. The addition of Addit or Codacide had no statistically significant effect on the number of viable spores of Vertalec sprayed per unit area (Tukey HSD test, *p* > 0.05).

In the laboratory bioassay against adult aphids, none of the EPF products caused aphid mortality by day three, but mortality had occurred by day six and it increased by day nine depending on the EPF product (Table 4). There was a significant effect of fungal treatment on aphid mortality at day six (*F* = 24.81, *d.f.* = 3, *p* < 0.001) and day nine (*F* = 13.94, *d.f*. = 3, *p* < 0.001). In post hoc tests, Botanigard ES caused significantly more mortality than the other products on both day six and day nine (Tukey HSD test, *p* < 0.001) and it was the only product to cause > 70% mean mortality in all aphid species (Table 4). Fungal treatment caused significantly more mortality in *B. brassicae* than in *N. ribisnigri* on both day six and day nine (Tukey HSD test, *p* < 0.001) but there was no significant difference in the percentage mortality of *N. ribisnigri* and *M. persicae*, or *B. brassicae* and *M. persicae.* At the end of the bioassay (day nine), Botanigard ES caused more mortality than Naturalis-L or Preferal WG in *M. persicae* and *N. ribisnigri* and Vertalec in *B. brassicae* (Tukey HSD tests, *p* < 0.05).

Application of the vegetable oil-based adjuvants Addit and Codacide caused no appreciable mortality in *M. persicae*, but Addit caused mortality in *B. brassicae* and *N. ribisnigri*, while Codacide caused mortality in *B. brassicae* (Table 4). For bioassays with *M. persicae* and *N. ribisnigri*, combining Vertalec with Addit, or Vertalec with Codacide, did not cause significantly different mortality compared to Vertalec on its own (Mann Whitney U test, *p* > 0.05). For *B. brassicae*, there was no significant difference in mortality caused by Vertalec, Addit or Codacide (Mann Whitney U test, *p* > 0.05), and combining Vertalec with Codacide did not result in greater mortality than Vertalec or Codacide on their own. In contrast, combining Vertalec with Addit caused significantly more *B. brassicae* mortality compared to Vertalec on its own (Mann Whitney U tests; day 6, *p* = 0.009; day 9, *p* = 0.029). The predicted mean mortality of the Vertalec + Addit combination, calculated from the fractional product of Vertalec and Addit mortality individually, was 42.4% (observed value = 44.0%) at day six, and 63.4% (observed value = 57.8%) at day nine.

There was significant variation in the number of progeny produced by the different aphid species both at day six (*F* = 400.46, *d.f.* = 2, *p* < 0.001) and day nine (*F* = 354.09, *d.f*. = 2, *p* < 0.001). There was also a significant variation in the number of progeny observed across the different EPF biopesticide treatments both at day six (*F* = 2.78, *d.f*. = 8, *p* = 0.009) and at day nine (*F* = 4.94, *d.f*. = 8, *p* < 0.001). All the EPF biopesticide products caused a reduction (*p* < 0.05) in the number of aphid progeny produced by *M. persicae* on day six and day nine compared to the control but there was no effect with *B. brassicae* or *N. ribisnigri* (*p* > 0.05) (Table 5). Overall the largest effects on *M. persicae* reproduction were observed with Botanigard ES, where the mean number of progeny produced per adult per day was reduced by 32% compared to the control by day six, and by 43% by day nine (Table 5). 

There was a significant effect of EPF treatment on the size of aphid populations that developed from nymphs treated with Botanigard ES and Preferal WG (*F* = 58.99, *d.f* = 2, *p* < 0.001). Treating aphid nymphs with both Botanigard ES and Preferal WG resulted in significantly smaller aphid populations seven and 10 days later compared to the control (*p* < 0.001) (Table 6). The effect on aphid population size was generally greater for Botanigard ES than for Preferal WG. However, neither EPF treatment resulted in elimination of the aphid populations. 

### 3.2. Field Experiments

In the first round of the field experiment (planted out 12th July), *B. brassicae* populations on untreated plants increased from a mean of 15.1 per plant at day seven to 162.8 per plant at day 21. There was no significant difference in the mean total number of aphids per plant (adults + nymphs) counted on Botanigard ES-treated plants and untreated plants seven days after the first spray (*F* = 2.18, *d.f*. = 1, *p* = 0.236) (Table 7). However, seven days after the second spray, there was a significant effect of the fungal biopesticide treatment (*F* = 12.01, *d.f.* = 1, *p* = 0.040), and aphid numbers were significantly lower on Botanigard ES-treated plants compared to untreated plants (equivalent to a 73% reduction in aphid population size) (Table 7).

A similar pattern was observed for the mean number of aphid nymphs per plant, with no significant effect seven days after the first spray, contrasted with a significantly lower number of nymphs on Botanigard ES-treated plants compared to untreated plants (*F* = 12.55, *d.f.* = 1, *p* = 0.038), again equivalent to a 73% reduction (Table 8). The mean temperature throughout the trial was 16.8 °C and ranged from 14 to 24 °C. There was 4.9 mm of rain and 5.2 h of sunshine per day (Table 9).

In the second round of field testing (planted out 16th August), the aphid populations remained low throughout the duration of the experiment (Table 10). There was a significant effect of fungal biopesticide treatment on the mean total number of aphids per plant (adults + nymphs) seven days after the first spray across all aphid species (*F* = 11.07, *d.f.* = 1, *p* = 0.045) but not at seven days after the second spray (*F* = 6.70, *d.f*. = 1, *p* = 0.081). Post hoc tests showed that Botanigard ES treatment resulted in significantly lower (*p* < 0.05) populations (adults + nymphs) of *B. brassicae* (54% reduction) and *N. ribisnigri* (88% reduction) compared to untreated plants seven days after the first spray; however, there was no significant effect on *M. persicae* (Table 10). This pattern was continued seven days after the second spray: post hoc tests showed that aphid populations were significantly lower (*p* < 0.05) for both *B. brassicae* (69% reduction) and *N. ribisnigri* (90% reduction) on Botanigard ES-treated plants compared to untreated controls, but with no significant effect on *M. persicae* populations (Table 10).

The same effects of Botanigard ES were observed for populations of nymphs in the field plots (Table 11). There was a significant effect of fungal biopesticide treatment on the mean number of nymphs per plant seven days after the first spray across all aphid species (*F* = 16.32, *d.f.* = 1, *p* = 0.027) and close to significant at seven days after the second spray (*F* = 8.83, *d.f*. = 1, *p* = 0.055). Post hoc testing showed that application of Botanigard ES caused no significant effect on nymph populations of *M. persicae* (*p* > 0.05). For *B. brassicae*, nymph populations were lower (*p* < 0.05) on Botanigard ES-treated plants compared to untreated controls both at seven days after the first spray (63% reduction) and seven days after the second spray (74% reduction). For *N. ribisnigri*, nymph populations were reduced by 63% in Botanigard ES-treated plots compared to untreated controls at seven days after the first spray (*p* < 0.05), and by 82% at seven days after the second spray (*p* < 0.05). The mean temperature throughout the trial was 13.8 °C and ranged from 10.3 to 20.1 °C. There was 1.6 mm of rain and 6.7 h of sunshine (Table 9).

## 4. Discussion

All three aphid species investigated in our laboratory bioassay were susceptible to infection by the four biopesticides tested. *Brevicoryne brassicae* showed more mortality than *N. ribisnigri* but no differences were detected between the susceptibilities of *N. ribisnigri* and *M. persicae*, or *B. brassicae* and *M. persicae*. Our results concur with other published work confirming the susceptibility of aphid pest species to EPF including *Akanthomyces*, *Beauveria*, *Cordyceps* and *Metarhizium* species [29,34,46,53,54,55,56,57,58]. Botanigard ES caused more mortality than the other biopesticide products in our bioassay. This is almost certainly because the product contained a higher concentration of spores than the other EPF biopesticides, resulting in a significantly higher dose (in terms of viable spores per mm^2^) when applied at the commercial rate. *Akanthomyces* species are reported to show greater inherent virulence than *Beauveria* and *Cordyceps* (*Isaria*) strains to aphids [46,59,60], but the dose of Botanigard ES (*B. bassiana* GHA) was 13 times higher than that of Vertalec (*A. dipterigenus* 1.72). It is likely that the ability to deliver a high spore dose with this product outweighed any issues with lower inherent virulence. It is also possible that differences in the formulations of the products affected their infectivity and virulence to aphids. 

There are few studies published with EPF where the applied dose is reported, in terms of spores per unit area, as a basis for understanding insect mortality [39]. This makes it difficult to compare results from different studies against the same target insect. For aphids, an exception is the work by Jandricic et al. [46], who evaluated strains of *B. bassiana*, *Metarhizium anisopliae* (Metchnikoff) Sorokin 1883 and *Cordyceps fumosorosea* s.l. in laboratory bioassays against *M. persicae*, as well as the melon cotton aphid, *Aphis gossypii* Glover, 1877, and the glasshouse potato aphid, *Aulacorthum solani* Kaltenbach, 1843. The bioassays were done using conidia suspensions produced from fungal cultures grown in the laboratory, as opposed to using commercial biopesticide preparations. They included fungal strains used in commercial products as well as non-commercial strains. In their study, *B. bassiana* GHA (Botanigard) and *C. fumosoroseus* s.l PFR97 (Preferal), performed poorly compared to *B. bassiana* JW-1 (Naturalis-L) and other, non-commercial, fungi. A dose of 2599 spores per mm^2^ of *B. bassiana* GHA resulted in 100% mortality of adult *M. persicae* after six days [46], compared to a dose of 640 spores per mm^2^ giving 80% mortality after seven days in our study. It is possible that our bioassay system would have required a markedly higher dose to achieve 100% mortality at day six, or it could be that the formulation used in the commercial preparation of *B. bassiana* GHA enabled aphids to be killed at a lower dose than compared to unformulated spores. Certainly, formulations are important for improving spray application characteristics, such as stability, mixing, and deposition [61]. In greenhouse experiments done prior to Vertalec being marketed to growers, it was found that the formulated product gave effective control of *A. gossypii* on a cucumber crop whereas unformulated spores did not give satisfactory control [62]. Oil-based formulations of EPF can also improve the efficacy of microbial biopesticides by increasing adhesion, spread and penetration of infective propagules on insect cuticle [63,64,65]. We found that two vegetable oil-based adjuvants, Addit and Codacide, could themselves cause mortality depending on the aphid species, with Addit causing mortality in *B. brassicae* and *N. ribisnigri*, and Codacide causing mortality in *B. brassicae*. Addit has been shown previously to cause moderate levels of mortality in tobacco whitefly, *Bemisia tabaci* Gennadius, 1889, and to be compatible with Naturalis (*B. bassiana* JW1), causing no inhibition of spore germination in a tank mix [66]. Vegetable oils can also give moderate control of aphids, which occurs by them smothering spiracles to prevent respiration, and they can improve the efficacy of low doses of chemical insecticides when applied as a combination [67,68]. However, in our study, there was no evidence that the adjuvants improved the effectiveness of EPF biopesticides in the laboratory bioassay, and in bioassays with *B. brassicae*, combining Addit and Vertalec had an additive effect. The use of proprietary rape seed oil-based adjuvants has been reported previously to have an additive effect on Vertalec against *M. persicae*, depending on the particular product used [69], and to improve the speed of kill of *M. anisopliae* against cattle ticks, *Boophilus microplus* Lahille 1905 [70]. 

An EPF biopesticide will only be effective if it can stop the pest population from reproducing, or at least lower the rate of reproduction down to agronomically acceptable levels. It can do this by: (i) killing adult insects so that the length of their reproductive period is shortened; (ii) causing pre-mortem reductions in adult reproductive rate; or (iii) killing juvenile stages before they reach reproductive age. Aphids are challenging to control because they have short development times to adulthood and high fecundity. For example, *M. persicae* is reported to complete development on *Brassica rapa* in 7.6 days at 23 °C [71], which gives only a relatively short window for EPF infection to kill immature stages of the pest before they reach reproductive age. We found a statistically significant reduction in the number of nymphs produced by *M. persicae* adults following EPF treatment, but no reduction was observed for *B. brassicae* or *N. ribisnigri.* There are differing reports in the literature on the effect of EPF infection on aphid reproduction, depending on the fungal species/strain and the aphid host. Jandricic et al. [46] observed limited effects on pre-mortem reproduction of *M. persicae*, *A. gossypii* and *A. solani* with *B. bassiana*, *M. anisopliae* and *C. fumosorosea* s.l., while no effects were observed for adult Russian wheat aphid, *Diuraphis noxia* Kurdjumov, 1913, infected with *B. bassiana* [72]. Similarly, infection of *M. persicae* with *Akanthomyces muscarius* (Petch) Spatafora, Kepler & B. Shrestha, comb. nov. (formerly *Lecanicillium muscarium* [32]) had no effect on reproductive rate but caused a significant decrease in the reproductive period, resulting in a decline in total fecundity, with the effect increasing with fungal dose [47]. In contrast, reduction in reproductive rate was observed for *B. bassiana* infection of pea aphid *Acyrthosiphon pisum* Harris, M., 1776 [53], in *Akanthomyces lecanii* (Zimm.) Spatafora, Kepler & B. Shrestha, comb. nov. (formerly *Lecanicillium lecanii* [32]) infection in wheat aphid *Schizaphis graminum* Rondani, 1852 [73], and in *B. bassiana* infection in *N. ribisnigri* [74].

Previous studies have reported that aphid nymphs are less susceptible to EPF infection than adults [46,74]. This is attributable to a short intermoult period, where moulting effectively removes any spores attached to the cuticle that have not yet penetrated the haemocoel, while the smaller size of nymphs can result in a lower spore dose being received [45]. In addition, lower levels of spore germination have been reported on nymphs compared to adults [45]. However, in our study, Botanigard ES and Preferal WG both had significant effects when applied against nymphs, and although the biopesticides did not eliminate the nymph populations, they caused a marked reduction for all aphid species with up to 86% reduction being observed (Botanigard ES applied to *M. persicae*, day 10).

Commercial EPF biopesticides have been shown to give significant reductions in aphid populations in greenhouse crops as part of an IPM approach [29,31]. For example, in greenhouse experiments on commercially grown lettuce, two spray applications of Vertalec caused significant reductions of *M. persicae*, *N. ribisnigri* and *M. euphorbiae*, with greater levels of control observed on *M. persicae* and *M. euphorbiae* than *N. ribisnigri* [34]. Few studies have been done to investigate EPF biopesticides against aphids under the more environmentally challenging conditions in field crops in temperate regions. In our first field experiment, two sprays of Botanigard ES, seven days apart, caused a 73% reduction in *B. brassicae* on cabbage plants, while in the second experiment, it caused a 69% reduction in *B. brassicae* and a 90% reduction in *N. ribisnigri*, but with no effect on *M. persicae* populations, and numbers of aphids remaining fairly small in controls. The half-life of activity of *B. bassiana* GHA to *N. ribisnigri* on lettuce foliage was estimated at six day in a semi-field experiment [74], which suggests that our spray application frequency was appropriate. The reasons for lack of control of *M. persicae* in our experiment are not known, but it may have been associated with the higher fecundity of *M. persicae* compared to *B. brassicae* and *N. ribisnigri* observed in the laboratory. Nevertheless, we consider these first results encouraging, and while Botanigard ES is unlikely to be used as a stand-alone treatment, we suggest that it has merit for further investigation as part of an IPM approach. Elsewhere, fungal strains that are virulent to aphids under controlled laboratory conditions have been found sometimes not to be effective in the field. For example, *B. bassiana* caused 100% mortality of hop aphids, *Phorodon humuli* Schrank, 1801, in the laboratory but did not give effective control in the field [75]. An aphid-derived strain of *B. bassiana* did not affect populations of *A. pisum* in field experiments in a lucerne crop in Oregon USA conducted in April and July, although the fungus persisted for at least 28 days after application [76]. The lack of control was attributed either to aphids not receiving a lethal dose in the field, or unsuitable temperature conditions, with temperatures varying between 6–20 °C in April and 10–25 °C in July [76]. The development of many EPF strains is reduced significantly below 16 °C which results in a slower speed of kill [39]. The optimum temperature for the growth of *B. bassiana* GHA, determined using a nonlinear model of poikilotherm development, is 29.8 °C [77]. In our field experiments, the average temperature was 16.8 °C for the first experiment, and 13.8 °C for the second experiment, suggesting that conditions were not optimal, but this did not stop the fungus from giving good levels of control of *B. brassicae* and *N. ribisnigri.* In field experiments in S Africa with *B. bassiana* GHA (used as the commercial product Mycotrol ES), fungal application caused 65% reduction in populations of *D. noxia* during a period with an average daily minimum of 10.2 °C and an average maximum of 23.9 °C [78]. Different species and strains of EPF can vary considerably in their thermal biology [26,39] and strains with cold tolerance have been reported which may be beneficial for use in temperate climates in the future [43,79,80], although future work is needed to better understand the effect of fluctuating field temperature on EPF performance, since it is possible that periods of warm daytime temperature could compensate partly for the inhibitory effect of cold night-time temperature. Other factors that affect field performance include humidity, rainfall (which can cause spores to be washed from foliar surfaces), solar radiation, and the architecture of the plant itself, which can affect the environmental conditions of the microclimate in the infection zone, as well as determining the density of spores deposited from spraying [39].

## 5. Conclusions

Aphids are economically important pests of field vegetables in temperate regions and are becoming increasingly difficult to control by conventional methods because of the declining availability of effective, synthetic chemical pesticides. The main aim of our paper was to investigate the potential of commercial fungal biopesticides, which are used normally against greenhouse pests, to help control *M. persicae*, *B. brassicae* and *N. ribisnigri* on brassica and lettuce in the field. At present, there is a lack of published information on the efficacy of fungal biopesticides against aphids on vegetable crops under field conditions. Our study suggests that Botanigard ES has potential for control of *B. brassicae* and *N. ribisnigri* on field vegetables. In order to get the best out of the biopesticide, future work should determine in more detail the effect of fungal infection on time to death of different instars and the impacts on reproductive rate, reproductive period, and population development. Research on the effects of fluctuating field temperatures on fungal performance is also warranted. Further insights into the mode of action of the fungus will enable spray timing and frequency of application to be optimized as part of Integrated Pest Management.

## Figures and Tables

**Table 1:** Aphid clones used in this study.

Species	Clone	Origin	Resistance Type
*Brevicoryne brassicae*	K3	Brussels sprouts, Lincolnshire, UK	-
*Myzus persicae*	4859B	Brussels sprouts, Yorkshire, UK	High esterase-R2, kdr-SR (heterozygote), MACE
*Myzus persicae* ^1^	2050A	Brussels sprouts, Lincolnshire, UK	Esterase-R2, kdr-SR, MACE
*Nasonovia ribisnigri*	4850A	Lettuce, Lincolnshire, UK	-

^1^ This clone was used in the field experiment.

**Table 2:** Crop protection products used in this study.

Product	Fungal Species	Manufacturer	Recommended Rate (Rate Used in Bioassay)
Botanigard ES	*Beauveria bassiana*	LAM International, USA	1.2–2.4 kg per 1000 L
			(2.44 mL in 1 L)
Naturalis-L	*Beauveria bassiana*	Biogard (CBC Europe), Italy	3 L/ha; 150 mL/100 L
			(4.4 mL in 1 L)
Preferal WG	*Cordyceps fumosorosea*	Biobest Group NV, Belgium	1–2 kg per 1000 L
			(2 g in 1 L)
Vertalec	*Akanthomyces dipterigenus*	Koppert Biological Systems, Netherlands	2 kg per 1000 L
			(2 g in 1 L)
Addit	-	Koppert Biological Systems, Netherlands	0.125–0.25%
			(2.5 mL in 1 L)
Codacide	-	Microcide Ltd., UK	2.5 L/ha
			(1 mL in 1 L)

**Table 3:** Mean number of colony forming units (cfus) per mm^2^ (±standard error of the mean) of EPF biopesticides applied to adult aphids in laboratory bioassays.

Treatment	Cfus per mm^2^ ± sem
Botanigard ES	639.6 ± 109.70
Naturalis-L	61.5 ± 7.59
Preferal WG	260.0 ± 72.48
Vertalec	50.8 ± 20.85
Vertalec + Addit	16.7 ± 4.46
Vertalec + Codacide	10.5 ± 4.21

**Table 4:** Susceptibility of three aphid species to entomopathogenic fungi (EPF) biopesticides in a laboratory bioassay. The data show the mean % mortality recorded at day 6 and day 9 after treatment (±the standard error of the mean).

	Day 6 % Mortality ± sem			Day 9 % Mortality ± sem		
	*M. persicae*	*B. brassicae*	*N. ribisnigri*	*M. persicae*	*B. brassicae*	*N. ribisnigri*
Botanigard ES	76.6 ± 6.74	84.3 ± 6.22	64.3 ± 6.64	79.5 ± 7.44	86.6 ± 6.59	71.0 ± 5.80
Naturalis-L	20.3 ± 5.85	48.4 ± 8.19	15.5 ± 4.08	40.2 ± 7.29	69.1 ± 9.50	27.0 ± 7.58
Preferal WG	38.1 ± 9.65	50.7 ± 5.70	34.6 ± 8.78	43.1 ± 10.38	60.8 ± 6.91	34.7 ± 8.99
Vertalec	51.4 ± 8.15	21.9 ± 7.06	33.2 ± 7.01	55.4 ± 8.29	43.8 ± 8.78	41.8 ± 7.70
Addit	3.2 ± 1.10	26.3 ± 7.04	16.3 ± 4.28	3.9 ± 1.44	34.8 ± 7.49	25.0 ± 5.75
Codacide	3.8 ± 1.49	17.4 ± 7.48	0.3 ± 0.35	1.7 ± 0.80	20.0 ± 5.74	3.1 ± 2.87
Vertalec + Addit	39.1 ± 7.41	44.0 ± 5.29	29.7 ± 7.75	52.9 ± 8.94	57.8 ± 5.73	33.9 ± 7.58
Vertalec + Codacide	39.0 ± 7.30	34.3 ± 12.41	36.7 ± 5.61	50.2 ± 11.77	36.5 ± 12.14	40.5 ± 6.73

**Table 5:** Mean number of aphid nymphs produced per adult per day (±standard error of the mean) following treatment of adult aphids with EPF biopesticides in a laboratory bioassay. The figures are calculated from the cumulative number of nymphs recorded at day 6 and day 9 after treatment of a starting cohort of 15 adult aphids.

Treatment	Day 6			Day 9		
	*M. persicae*	*B. brassicae*	*N. ribisnigri*	*M. persicae*	*B. brassicae*	*N. ribisnigri*
Control	9.3 ± 0.66	3.7 ± 0.42	1.4 ± 0.26	8.2 ± 0.61	3.1 ± 0.37	1.3 ± 0.25
Botanigard ES	6.3± 0.55	3.1 ± 0.38	0.8 ± 0.20	4.6 ± 0.45	2.1 ± 0.31	0.6 ± 0.17
Naturalis L	7.2 ± 0.58	2.8 ± 0.36	1.4 ± 0.26	6.3 ± 0.53	2.6 ± 0.34	1.2 ± 0.23
Preferal WG	6.3 ± 0.55	3.4 ± 0.40	0.9 ± 0.21	5.5 ± 0.50	2.5 ± 0.34	1.0 ± 0.21
Vertalec	7.0 ± 0.58	3.9 ± 0.43	1.3 ± 0.25	5.6 ± 0.50	3.1 ± 0.37	1.2 ± 0.23
Addit	7.5 ± 0.69	4.0 ± 0.43	1.2 ± 0.23	7.1 ± 0.66	3.1 ± 0.37	1.1 ± 0.22
Codacide	8.0 ± 0.96	3.1 ± 0.47	1.5 ± 0.41	7.7 ± 0.93	2.5 ± 0.42	1.4 ± 0.38
Vertalec + Addit	6.8 ± 0.57	3.2 ± 0.39	1.0 ± 0.22	5.9 ± 0.51	2.4 ± 0.33	1.0 ± 0.21
Vertalec + Codacide	7.0 ± 0.89	3.8 ± 0.52	1.7 ± 0.44	6.3 ± 0.84	2.8 ± 0.44	1.4 ± 0.39

**Table 6:** Results of a laboratory bioassay of EPF biopesticides against aphid nymphs of *M. persicae*, *B. brassicae* and *N. ribisnigri*. Groups of 15 nymphs were treated with biopesticides and then left to develop into adults and reproduce. Data represent the mean total number of aphids per plant counted at 7 and 10 days after EPF biopesticide application (±standard error of the mean).

Treatment	Day 7			Day 10		
	*M. persicae*	*B. brassicae*	*N. ribisnigri*	*M. persicae*	*B. brassicae*	*N. ribisnigri*
Control	34.6 ± 3.71	22.4 ± 2.92	28.0 ± 3.71	65.0 ± 6.11	54.4 ± 5.58	26.1 ± 4.29
Botanigard	8.5 ± 1.86	4.1 ± 1.21	6.7 ± 1.72	9.1 ± 2.34	6.3 ± 1.79	13.9 ± 3.01
Preferal	20.3 ± 2.76	8.4 ± 1.76	9.6 ± 2.09	36.7 ± 4.46	13.7 ± 2.69	7.3 ± 2.21

**Table 7:** Mean total number of *B. brassicae* recorded per cabbage plant after two treatments with Botanigard ES (*B. bassiana*) in field experiment 1 (square root-transformed data are given in parentheses). Applications of Botanigard ES were done on days 7 and 14 after inoculation of plants with aphids. Counts of aphid numbers were done immediately prior to spraying on day 7 and day 14, and 7 days after the second spray (=day 21).

	Pre-Spray (Day 7)		Post Spray 1 (Day 14)		Post Spray 2 (Day 21)	
	Untreated	Treated	Untreated	Treated	Untreated	Treated
Count	15.1 (3.93)	13.1 (3.67)	64.3 (8.04)	47.3 (6.91)	162.8 (12.77)	43.8 (6.64)
	(sed 0.468, 3 *d.f.*)		(sed 0.770, 3 *d.f.*)		(sed 1.769, 3 *d.f.*)	

**Table 8:** Mean total number of *B. brassicae* nymphs recorded per cabbage plant after two treatments with Botanigard ES in field experiment 1 (square root-transformed data are given in parentheses). Counts of aphid numbers were done immediately prior to spraying on day 7 and day 14, and 7 days after the second spray (=day 21).

	Pre-Spray (day 7)		Post Spray 1 (day 14)		Post Spray 2 (day 21)	
	Untreated	Treated	Untreated	Treated	Untreated	Treated
Count	12.9 (3.65)	11.0 (3.37)	49.3 (7.05)	35.8 (6.02)	135.2 (11.64)	35.9 (6.03)
	(sed 0.447, 3 *d.f.*)		(sed 0.723, 3 *d.f.*)		(sed 1.589, 3 *d.f.*)	

**Table 9:** Mean environmental conditions (±standard error of the mean) in field experiment 1 and 2.

	Experiment 1	Experiment 2
Temperature (°C)	16.8 ± 0.43	13.8 ± 0.44
Maximum temperature (°C)	24.0 ± 0.33	20.1 ± 0.62
Minimum temperature (°C)	14.0 ± 0.44	10.3 ± 0.51
Rainfall (mm)	4.9 ± 1.49	1.6 ± 0.47
Sunshine (h)	5.2 ± 0.69	6.7 ± 0.60
Relative humidity (%)	75.3 ± 1.66	76.7 ± 1.64

**Table 10:** Mean total number of *M. persicae*, *B. brassicae*, and *N. ribisnigri* recorded per plant after two treatments with Botanigard ES in field experiment 2 (square root-transformed data are given in parentheses). Applications of Botanigard ES were done on days 7 and 14 after inoculation of plants with aphids. Counts of aphid numbers were done immediately prior to spraying on day 7 and day 14, and 7 days after the second spray (=day 21).

	Pre-Spray (Day 7)		Post Spray 1 (Day 14)		Post Spray 2 (Day 21)	
	Untreated	Treated	Untreated	Treated	Untreated	Treated
*M. persicae*	13.8 (3.76)	18.1 (4.30)	9.6 (3.16)	8.0 (2.90)	11.2 (3.41)	9.1 (3.08)
*B. brassicae*	28.3 (5.35)	25.1 (5.05)	23.0 (4.83)	10.5 (3.30)	47.4 (6.91)	14.7 (3.88)
*N. ribisnigri*	3.9 (2.08)	2.6 (1.74)	4.3 (2.16)	0.5 (0.96)	7.0 (2.72)	0.7 (0.53)
sed (*p* < 0.05; *d.f.* = 3): untreated—treated	0.157		0.300		0.662	
sed (*p* < 0.05; *d.f.* = 12): aphid—aphid	0.272		0.459		0.872	

**Table 11:** Mean total number of *M. persicae*, *B. brassicae*, and *N. ribisnigri* nymphs recorded per plant after two treatments with Botanigard ES in field experiment 2 (square root-transformed data are given in parentheses). Applications of Botanigard were done on days 7 and 14 after inoculation of plants with aphids. Counts of aphid numbers were done immediately prior to spraying on day 7 and day 14, and 7 days after the second spray (=day 21).

	Pre-Spray (Day 7)		Post Spray 1 (Day 14)		Post Spray 2 (Day 21)	
	Untreated	Treated	Untreated	Treated	Untreated	Treated
*M. persicae*	9.8 (3.20)	14.5 (3.86)	7.3 (2.76)	6.0 (2.53)	8.0 (2.89)	5.2 (2.36)
*B. brassicae*	23.9 (4.93)	21.5 (4.68)	16.2 (4.08)	6.0 (2.53)	33.8 (5.85)	8.7 (3.02)
*N. ribisnigri*	1.2 (1.09)	0.9 (0.96)	2.4 (1.67)	0.6 (0.77)	3.3 (1.92)	0.6 (0.78)
sed (*p* < 0.05; *d.f.* = 3): untreated—treated	0.066		0.221		0.505	
sed (*p* < 0.05; *d.f.* = 12): aphid—aphid	0.291		0.375		0.724	
